# ATP Release and Purinergic Signaling Potentiate β‐Adrenergic Effects on Brown Adipocytes

**DOI:** 10.1111/apha.70307

**Published:** 2026-09-07

**Authors:** Marco Tozzi, Emilie B. Berggreen, Rui Zhang, Anders Gudiksen, Wei Li, Mathias B. Matz, Henriette Pilegaard, Jacob B. Hansen, Ivana Novak

**Affiliations:** ^1^ Section for Cell Biology and Physiology, Department of Biology University of Copenhagen Copenhagen Denmark

**Keywords:** adipocyte, brown adipose tissue, extracellular ATP, glycolysis, lipolysis, oxygen consumption, P2X receptor expression, pannexin channels, thermogenesis

## Abstract

**Aim:**

Brown adipose tissue (BAT) specializes in energy consumption and thermogenesis in response to cold stress. Brown adipocytes (BA) express purinergic receptors, yet little is known about the source of extracellular ATP and the role of purinergic signaling, and the potential interaction with β‐adrenergic signaling. We aimed at elucidating whether: BA could release extracellular ATP; ATP/purinergic signaling impacts β‐adrenergic responses; and the expression of purinergic receptors in BAT of mice and cultured BA.

**Methods:**

Real‐time ATP release was monitored in a mature BA cell model exposed to adrenergic agonists, insulin, glucose, and pharmacological inhibitors. Cellular assays included oxygen consumption, glycolysis, and lipolysis assays, and Ca^2+^ imaging. The protein level of ATP‐release protein, pannexin‐1, was determined. RNA‐sequencing was performed on mouse BAT and cultured BA.

**Results:**

We show that β‐adrenergic stimulation of BA causes ATP release through pannexin‐1 in a cAMP‐protein kinase A‐dependent manner. ATP release is increased with glucose but decreased with insulin, the latter due to phosphodiesterase 3 activation. β‐Adrenergic stimulation increases oxygen consumption, glycolysis, and lipolysis, while apyrase and pannexin‐1 inhibitors decrease these effects. Both extracellular ATP and β‐adrenergic stimulation induce Ca^2+^ signals, albeit with different kinetics. BAT from mice exposed to cold and BA treated with norepinephrine show regulation of several purinergic receptors.

**Conclusions:**

The present study demonstrates that ATP is released from BA via pannexin‐1 channels in response to β‐adrenergic stimulation, that extracellular ATP, acting through P2 receptor signaling, potentiates β‐adrenergic effects on BA function, and that expression of several purinergic receptors changes in response to β‐adrenergic stimulation.

## 1 Introduction

Brown adipose tissue (BAT) is specialized in energy expenditure by dissipating chemical energy as heat through non‐shivering thermogenesis in response to environmental cues such as cold exposure. Brown adipocytes (BA) have a high density of mitochondria, multilocular lipid droplets and high expression of uncoupling protein‐1 (UCP1), which is the predominant mediator of non‐shivering thermogenesis. UCP1 is embedded in the inner mitochondrial membrane and uncouples the electron transport system from ATP synthesis by facilitating proton leak from the intermembrane space back into the mitochondrial matrix. The energy is dissipated as heat because there is no biochemical reaction to accept the released energy from the proton leak through UCP1 [1].

The main regulators of uncoupling in BAT are the sympathetic nerves releasing norepinephrine, activating β‐adrenergic receptors on the surface of BA. This results in cAMP/protein kinase A (PKA) signaling, upregulation and activation of UCP1, phosphorylation of protein kinase B (Akt) and hormone‐sensitive lipase (HSL), together leading to increased thermogenesis, mitochondrial biogenesis, lipolysis and fatty acid oxidation, as well as glucose uptake and metabolism [1, 2, 3].

It is presumed that sympathetic nerves release not only norepinephrine but also ATP [4, 5], and that ATP, and its hydrolytic and conversion products such as ADP, adenosine, UTP and UDP, could affect adipocyte function via purinergic signaling [6, 7, 8]. In white adipose tissue (WAT), adipocytes release ATP upon α‐adrenergic stimulation [9, 10], and purinergic P2 receptors co‐regulate white adipocyte function, including effects on WAT inflammation [6, 9]. Recently, the potential role of purinergic signaling in regulating BAT function has become an active field of research [6, 11]. One study showed that ATP and adenosine are released from sympathetic nerves in BAT, but that BA also released adenosine [8]. Another study demonstrated that BA express the ATP release channel, pannexin‐1 (Panx1), that influences adaptive thermogenesis [12]. However, the authors suggested that these Panx1 effects were independent of ATP release and purinergic signaling. Since one study reported that mouse and human BA have a high expression of the P2X5 receptor that parallels the expression of UCP1, the P2X5 receptor was suggested as a BA marker [13]. Therefore, recent studies have focused on this and other P2 receptors. Whole‐body and BA‐specific knockout of *P2rx5* caused reduced levels of UCP1 protein in mouse BAT and led to impaired glucose tolerance without impacting systemic energy expenditure [14]. Another study demonstrated that P2X5 receptor‐deficient mice displayed reduced browning and higher energy expenditure at room temperature and upon cold challenge [15]. Wild‐type and β‐adrenergic receptor‐deficient mice housed at room temperature gained and lost, respectively, fat mass in response to chronic subordination stress [5]. Under such conditions, mice lacking β‐adrenergic receptors demonstrated increased levels of UCP1 accompanied by increased expression of P2X7 and P2Y_12_ receptors in BAT, suggesting an involvement of purinergic signaling in the regulation of energy expenditure independent of β‐adrenergic effects [5]. In diet‐ or genetically induced obese mice, expression of P2X7 receptors (and P2X4 and P2X5) was increased in WAT and BAT, but the P2X7 receptor was dispensable for regulation of energy expenditure [16]. Furthermore, the P2Y_6_ receptor was shown to negatively regulate adipose tissue browning in mice [17]. Thus, functional studies in mice focusing on different P2 receptors, for example, ATP‐gated ion channels with different affinities for ATP such P2X4, P2X5, P2X7 receptors, and G‐protein coupled receptors with higher affinity for other nucleotides such as ADP‐sensitive P2Y_6_ and UDP‐sensitive P2Y_12_ receptors, show divergent outcomes. Since BAT is comprised of several cell types (adipocytes, vascular, immunoreactive and nerve cells), one will need to understand P2R distribution and interplay between BAT cells, and not least, pinpoint the sites/cells and conditions that lead to physiological as opposed to pathophysiological (stress) ATP release. Here, we focus on the central BAT cell, the brown adipocyte.

We hypothesize that BA release ATP under physiological conditions and that extracellular ATP (eATP) can, via purinergic signaling, contribute to the regulation of basic BA functions such as oxygen consumption, glycolysis, and lipolysis. The present study aimed to elucidate whether BA release ATP, how this process is regulated, and whether purinergic signaling is coordinated with β‐adrenergic signaling. Furthermore, RNA‐sequencing was applied to BA and BAT to assess the dynamics of P2 receptor expression in response to β‐adrenergic stimulation.

## 2 Results

### 2.1 Brown Adipocytes Release ATP Through Pannexin‐1 Upon β‐Adrenergic Stimulation

**FIGURE 1:**
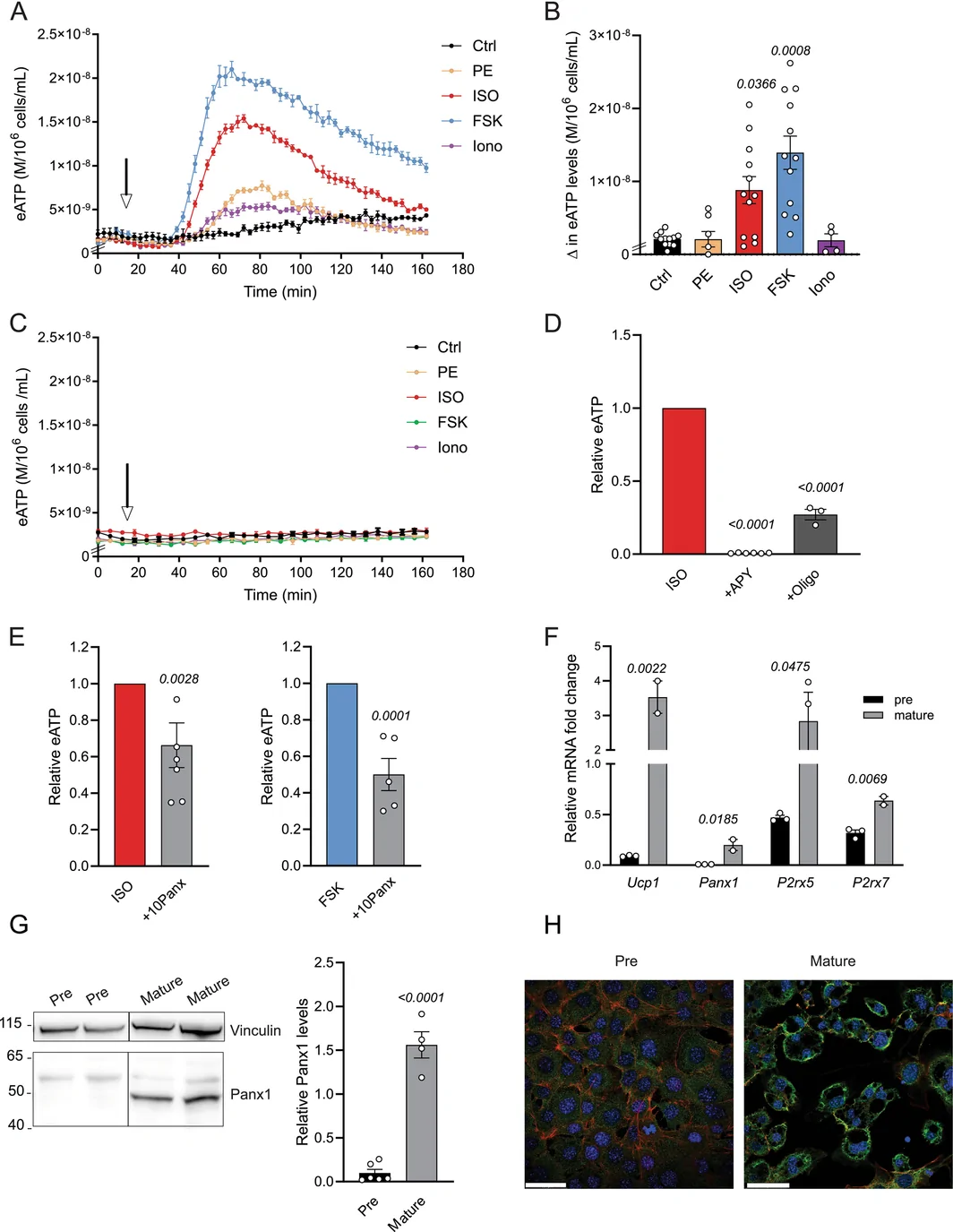
Adrenergic stimulation induces ATP release in mature brown adipocytes. (A) Representative graph of the eATP concentrations with time in mature brown adipocytes BA, that is, WT‐1 cells, upon stimulation (arrow) with control buffer (Ctrl), 5 μM phenylephrine (PE), 1 μM isoproterenol (ISO), 10 μM forskolin (FSK) and 1 μM ionomycin (Iono), as indicated. Data represent means ± SEM of three technical replicates in one representative experiment. (B) Quantification of eATP in mature adipocytes is shown as a difference between the basal eATP (before stimulation) and the peak release with the indicated agonist. The graph shows individual data and means ± SEM from 4 to 12 independent experiments. Data were log transformed before statistical analysis was performed, and numbers indicate significant *p* values compared to Ctrl. (C) Representative graph of eATP concentrations with time in premature adipocytes. Similar notations as in A. (D) Effect of 5 U/mL apyrase (APY), and 1 μM oligomycin (Oligo) on ISO‐induced ATP release. (E) ISO‐ and FSK‐induced ATP release from mature adipocytes pre‐treated with 100 μM pannexin‐1 inhibitor ^10^Panx (10Panx). (D, E) Normalized data, though first quantified with respect to cell numbers and chamber volumes (see Methods), numbers indicate significant *p* values compared to respective controls. (F) Relative RNA of WT‐1 pre‐ and mature adipocytes. Data are represented as means ± SEM, and *p* values are given. (F) Relative expression of mRNA for *Ucp1* (control marker), *Panx1* and *P2rx5* and *P2rx7* in pre‐ and mature adipocytes. (G) Representative western blot and quantification of 45 kDa Panx1 protein levels (Panx1/vinculin) in pre‐ and mature adipocytes. This blot is a part of a larger blot where other cells and protocols were tested. (H) Localization of pannexin‐1 (green), F‐actin (red) and nuclei (blue) in adipocytes in premature (left image) and mature (right image) adipocytes. The images are representative of 4 independent staining experiments. The bar is 50 μm.

The first objective was to determine whether BA are capable of regulated ATP release. For this purpose, similar to our study on WA [9], we set up an assay for monitoring eATP on‐line on living cells with appropriate corrections for cell numbers and chamber volumes. Mature WT‐1 brown adipocytes were stimulated with different agents: α‐ and β‐adrenergic agonists (phenylephrine and isoproterenol (ISO)), adenylyl cyclase activator (forskolin), or calcium ionophore (ionomycin), and the extracellular ATP concentration (eATP) was monitored in real‐time (Figure 1A). Adrenergic β‐receptor stimulation with ISO induced a robust and regulated increase in eATP in mature BA, peaking approximately 20–30 min after stimulation. This eATP increase was most likely due to an increase in ATP release (see results and discussion below). Forskolin also induced a robust increase in eATP with similar kinetics, while α‐adrenergic receptor stimulation with phenylephrine yielded much smaller effects on eATP (Figure 1A,B). Ionomycin, which induced Ca^2+^ influx (data not shown), and the control solution (5.5 mM glucose buffer) had minimal impact on eATP levels (Figure 1A,B). Interestingly, pre‐adipocytes (undifferentiated WT‐1 cells) did not show any increase in eATP upon either adrenergic, forskolin, or ionomycin stimulation (Figure 1C). Next, we investigated the effect of ATP‐modulating agents on eATP (Figure 1D). Apyrase, which hydrolyses ATP (and ADP), and oligomycin, which inhibits mitochondrial ATP formation, both decreased isoproterenol‐induced eATP levels (Figure 1D).

The second objective was to investigate whether the increase in eATP was due to ATP release through Panx1, and hence the effect of the Panx1 inhibitor (^10^Panx) was tested. As shown in Figure 1E, both isoproterenol‐ and forskolin‐induced ATP release were significantly diminished by ^10^Panx. Next, the expression of Panx1 in pre‐adipocytes and mature adipocytes was assessed at the mRNA and protein levels (Figure 1F,G). Relative mRNA for Panx1 was increased in mature adipocytes. Also, like UCP1 mRNA, levels of P2X5 and P2X7 receptor mRNAs increased in mature adipocytes (Figure 1F and see below). Representative western blots showed that the anti‐Panx1 antibody recognized two bands at ~45 and ~48 kDa in both pre‐ and mature BA and quantification of the two bands across independent experiments showed that the content of ~45 kDa protein was markedly upregulated in the mature state (Figure 1G). Immunolocalization of Panx1 was detected in the cell periphery of mature adipocytes (Figure 1H, right image), while pre‐adipocytes showed weak and diffuse staining, and more prominent F‐actin fibers (Figure 1H, left image).

### 2.2 ATP Release Depends on cAMP/PKA Signaling and Is Sensitive to Glucose and Insulin

**FIGURE 2:**
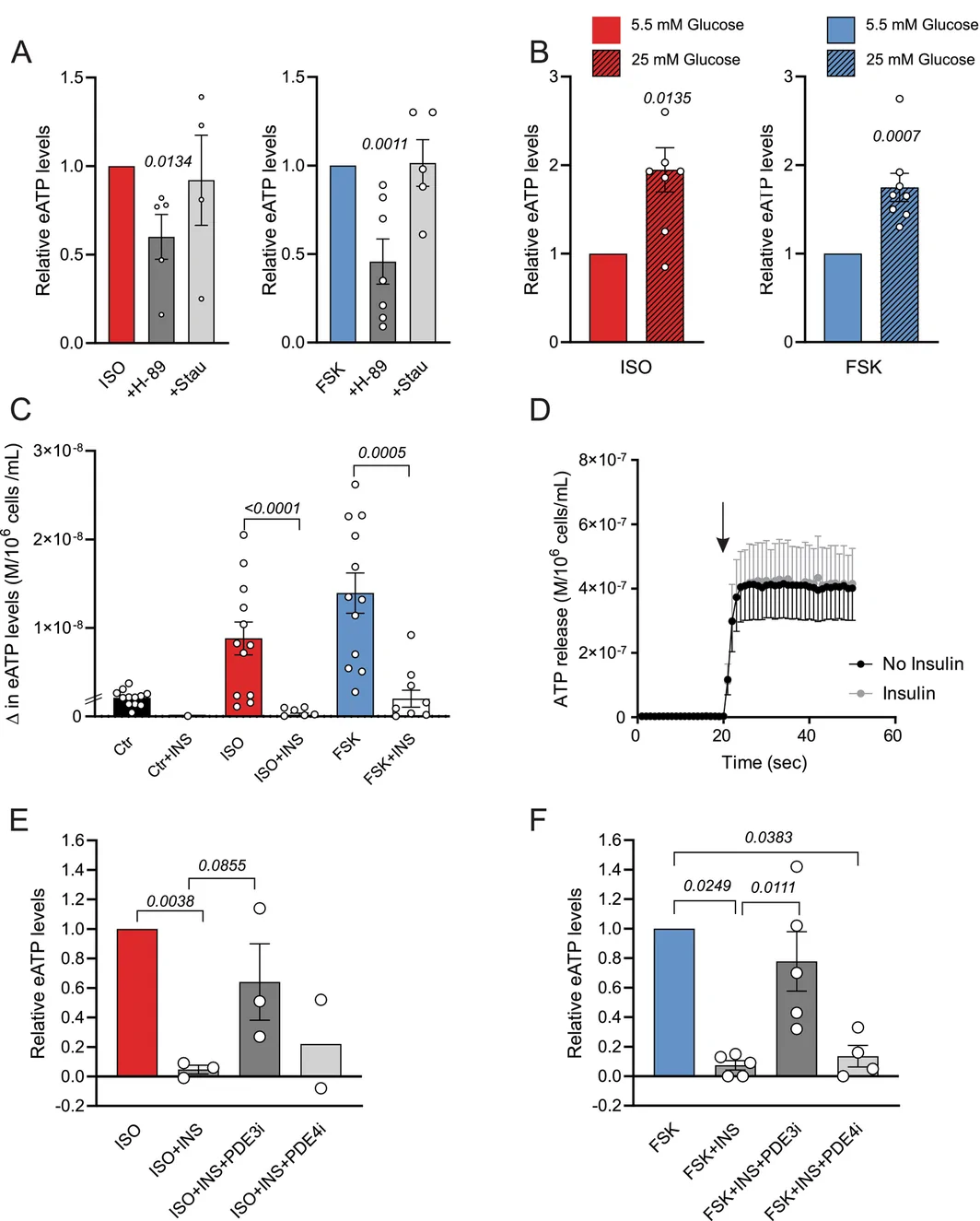
Effect of protein kinases, glucose, insulin and phosphodiesterases on ATP release in mature brown adipocytes. (A) The effect of 20 μM PKA inhibitor H‐89 and 10 nM PKC inhibitor staurosporine (Stau) on normalized eATP levels induced by ISO or FSK (see Methods). (B) Normalized eATP levels in mature adipocytes cultured for 24 h in low (5.5 mM) or high (25 mM) glucose concentrations. (C) Quantified eATP levels (peak‐basal ATP) in unstimulated cells, Ctr (black bar), following stimulation with 1 μM ISO and 10 μM FSK in the presence or absence of 1 μg/mL insulin (INS) (white bar). (D) Insulin does not affect eATP levels induced by mechanical stimulation (pump injection of physiological buffer indicated by an arrow). Data shows means ± SEM for 3 independent experiments. (E, F) Normalized eATP levels induced by ISO (E) or FSK (F) in the presence of insulin and either 50 μM cilostazol, PDE3 inhibitor (PDE3i), or 20 μM rolipram, PDE4 inhibitor (PDE4i). Significance levels are indicated in the graphs. Abbreviations are the same as in Figure 1.

The next objective was to consider physiological factors and signaling pathways regulating ATP release. First, we assessed whether PKA or protein kinase C (PKC) was involved in the signaling pathway mediating the adrenergically‐induced ATP release. Inhibition of PKA with the selective inhibitor H‐89 impaired both the isoproterenol‐ and forskolin‐stimulated increase in eATP, while inhibition of PKC by staurosporine had no significant effects on stimulated eATP levels (Figure 2A).

In the following experiments, we tested whether glucose availability and/or metabolism might affect eATP levels. Cells incubated for 24 h with high glucose concentration (25 mM) and then stimulated with isoproterenol and forskolin had significantly enhanced eATP compared to cells incubated with physiological/control glucose concentration (5.5 mM) (Figure 2B). Given the impact of glucose, and given the insulin sensitivity of adipocytes, we investigated whether insulin could affect eATP. Remarkably, pre‐treatment with insulin (1 μg/mL) markedly reduced isoproterenol‐ and forskolin‐induced increases in eATP (Figure 2C). In contrast, insulin had no effects on mechanically induced ATP release, which probably involves other pathways than Panx1 (Figure 2D).

A downstream effect of insulin receptor stimulation in insulin‐sensitive cells, like adipocytes, is the protein kinase B (PKB/Akt)‐dependent phosphorylation and inactivation of cyclic nucleotide phosphodiesterases (PDEs) that hydrolyse cAMP [18]. BA express mainly PDE3 and PDE4, and both are key players in insulin‐regulated lipolysis, lipogenesis, and glucose uptake [19, 20]. Therefore, we tested whether PDE3 and/or PDE4 were involved in the insulin‐mediated inhibition of ATP release. Indeed, inhibition of PDE3 by cilostazol restored forskolin‐induced ATP release in the presence of insulin (Figure 2F). For the isoproterenol‐induced effect on eATP, although a multiple comparison of groups did not show a significant difference, pairwise tests showed significant inhibition by insulin (ISO ± insulin, *p* = 0.0038) while inhibition of PDE3i (ISO + insulin ± PDE3i, *p* = 0.0855) rescued the ISO effect. In contrast, PDE4 inhibition by rolipram did not prevent the inhibitory effects of insulin on ATP release (Figure 2E,F).

### 2.3 Role of β‐Adrenergic Stimulation and eATP on Oxygen Consumption in Brown Adipocytes

**FIGURE 3:**
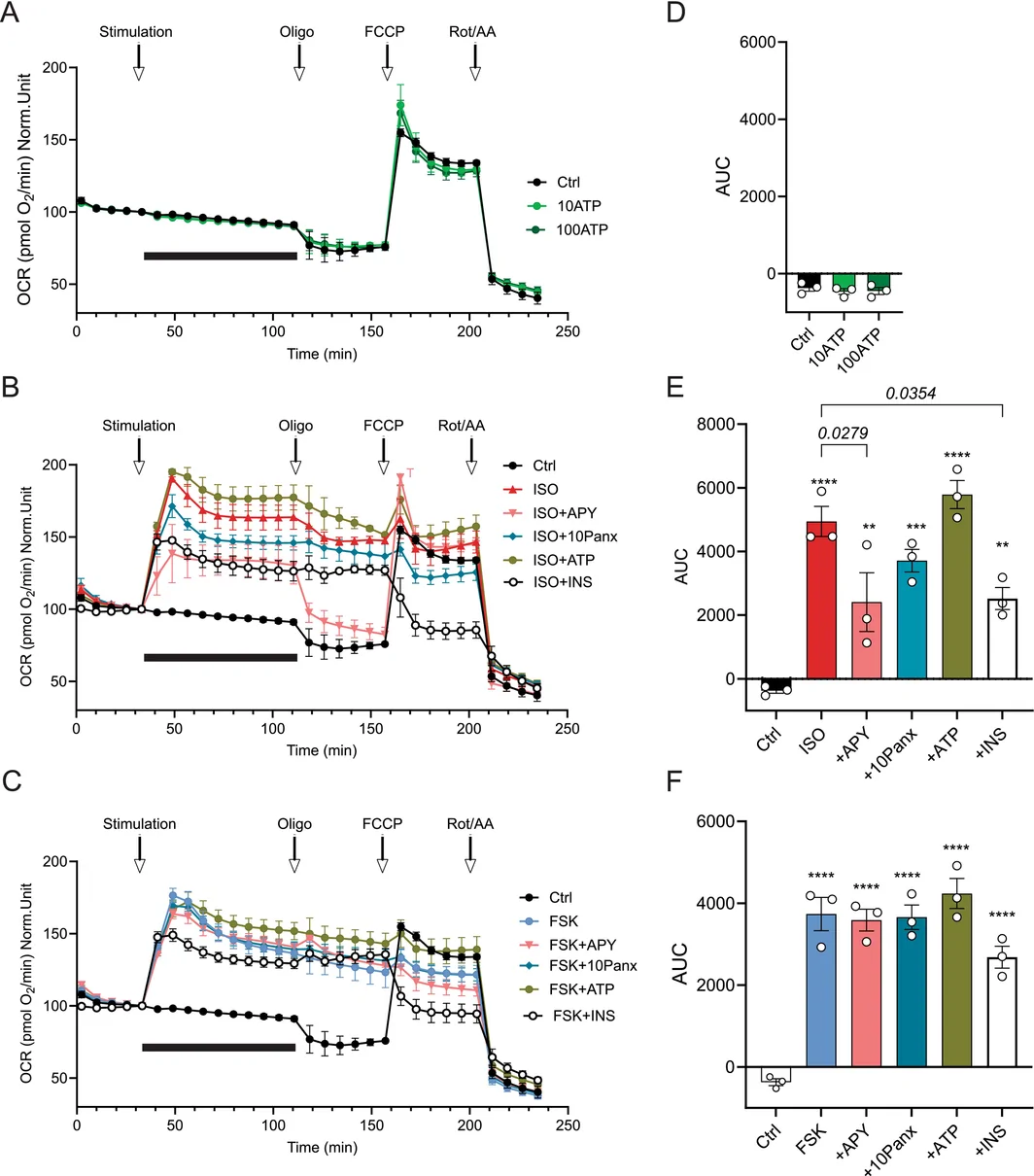
OCR in mature brown adipocytes and BAT in response to β‐adrenergic and ATP stimulation. OCR in mature BA (WT‐1 cells) was measured on the Seahorse XF96 Analyzer under basal conditions; then cells were stimulated by the addition of Seahorse media (Ctrl) and the following: (A) 10 μM or 100 μM ATP; (B) 1 μM ISO or ISO in combination with 100 μM ATP; (C) 10 μM FSK or FSK in combination with 100 μM ATP. Some cells (B, C) were pretreated with 5 U/mL apyrase (APY), or 100 μM ^10^Panx or 1 μg/mL insulin (INS) for 45 min before stimulation. At the end of the experiment, cells were treated with 1 μM Oligo, 0.5 μM FCCP, and 1 μM Rot/AA (see Methods), as indicated. (A–C) OCR measurements shown as mean values ± SEM of 3 independent experiments (with 4–6 replicates per experiment). Readings were normalized to the stimulant injection point, defined as the baseline. (D–F) Area under the curve (AUC) of OCR after stimulation (period indicated by the bars in A–C) corrected for the baseline. Bar graphs represent data for each experiment, and mean values ± SEM. Asterisks depict significant differences between the control and tested compounds (****p < 0.0001, ***p < 0.005, **p < 0.01). *p* values show significant differences between ISO stimulation and ISO + test compounds.

In the next series of experiments, we probed the physiological role of eATP released from BA following adrenergic stimulation. In some experiments, we added exogenous eATP. Since we have shown that ATP release is linked to cell metabolism in white adipocytes (WA) [9], it was therefore relevant to investigate whether the same applied to BA. It is known that activation of β‐adrenergic receptors elevates BA respiration [1], and our experiments (see above) showed that β‐adrenergic and PKA/adenylyl cyclase stimulation induced ATP release. Therefore, we tested whether eATP influences BA energy metabolism. Mitochondrial function was assessed as oxygen consumption rate (OCR) using a Seahorse analyzer (Figure 3). Addition of exogenous ATP on its own (10 or 100 μM) had no significant effect on OCR (Figure 3A,D). However, pre‐treatment with apyrase (to hydrolyse ATP) significantly decreased ISO‐stimulated OCR (Figure 3B,E). Pre‐treatment with Panx1 inhibitor tended to reduce isoproterenol effects, although statistical significance was not obtained. Pre‐treatment with exogenous ATP of cells incubated with isoproterenol had no additional effects on OCR (Figure 3B,E). When adipocytes were stimulated with forskolin instead of isoproterenol, OCR was also increased, but compounds affecting eATP (see Figure 1) had no significant effect (Figure 3C,F). Effects of metabolic modulators oligomycin and FCCP were most pronounced in non‐stimulated control cells and stimulated cells treated with apyrase. Notably, insulin pre‐treatment reduced isoproterenol‐stimulated OCR but only tended to decrease forskolin‐induced OCR (Figure 3E,F). However, basal OCR, just prior to the time of stimulation was significantly increased by treatment with insulin from 158 ± 5 to 270 ± 7 (means ± SEM, *n* = 6, *p* < 0.0001) (data not shown).

To validate our Seahorse OCR results, we performed high‐resolution respirometry with Oxygraph 2 k on interscapular BAT (iBAT) explants dissected from mice. Stimulation with isoproterenol or forskolin and the addition of ATP agents had small and variable effects (data not shown). We reasoned that the applied compounds might not permeate into the tissue pieces and that handling of the tissue during dissection and in the experimental chamber would release ATP. Therefore, this experimental set‐up may not be suited for studying purinergic signaling, and we did not pursue these experiments further.

### 2.4 Role of β‐Adrenergic Stimulation and eATP in Glycolysis in Brown Adipocytes

**FIGURE 4:**
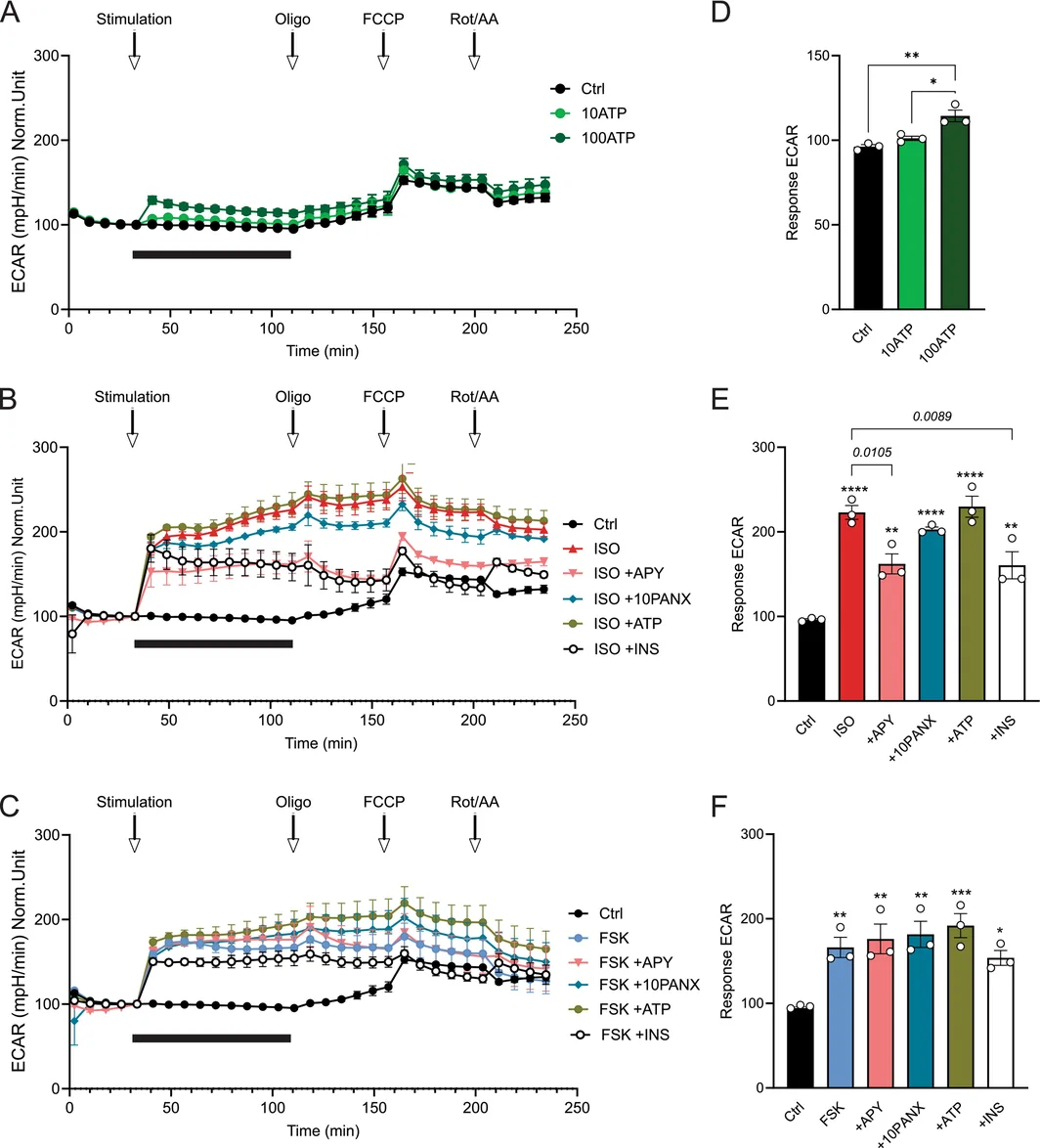
ECAR in mature brown adipocytes in response to ATP and β‐adrenergic stimulation. ECAR was measured on the Seahorse XF96 Analyzer under basal conditions; then cells (WT‐1) were stimulated by the addition of Seahorse media (Ctrl) containing: (A) 10 μM or 100 μM ATP; (B) 1 μM ISO or ISO in combination with 100 μM ATP; (C) 10 μM FSK or FSK in combination with 100 μM ATP. Some cells were pretreated with 5 U/mL APY, 100 μM ^10^Panx or 1 μg/mL insulin (INS) for 45 min before stimulation. At the end of the experiment, cells were treated with 1 μM Oligo, 1 μM FCCP, and 1 μM Rot/AA (see Methods), as indicated. (A–C) ECAR measurements are given as means ± SEM of 3 independent experiments (with 4–6 replicates per experiment). Readings were normalized to the stimulant injection point. (D–F) Mean ECAR responses after stimulation. Data are represented for each experiment together with means ± SEM. *p* values for tests between control and ATP are given in A. For clarity, in B and C, asterisks depict significant differences between the control and tested compounds (****p < 0.0001, ***p < 0.0005, **p < 0.005) while *p* values show significant differences between ISO stimulation and ISO + test compounds.

In addition to oxygen consumption, we also measured extracellular acidification as an indicator of glycolysis and lactic acid secretion, and possibly other acid‐extruding processes (see Discussion). Extracellular acidification rate (ECAR) was monitored by the Seahorse analyzer. The experimental set‐up for ECAR was like the one used for OCR (Figure 4). Interestingly, adding exogenous ATP alone increased ECAR in a dose‐dependent manner, although effects were modest (Figure 4A,D). ISO significantly increased ECAR, and this effect was attenuated by pre‐treatment with apyrase and insulin (Figure 4B,E). Pre‐treatment with Panx1 inhibitor tended to reduce ISO effects, although statistical significance was not reached. Forskolin also stimulated ECAR, but none of the compounds tested significantly altered this forskolin effect (Figure 4C,F). Again, effects of oligomycin (to upregulate glycolysis) and FCCP (to upregulate uncoupled cellular respiration) were most notable in non‐stimulated control cells. Like OCR (Figure 3E,F), insulin reduced ISO‐stimulated ECAR (Figure 4C,F). However, basal ECAR was significantly increased from 19.2 ± 0.2 to 66.0 ± 3.1 by insulin alone (*n* = 6, *p* < 0.0001) (data not shown).

### 2.5 Role of eATP on β‐Adrenergically Stimulated Lipolysis

**FIGURE 5:**
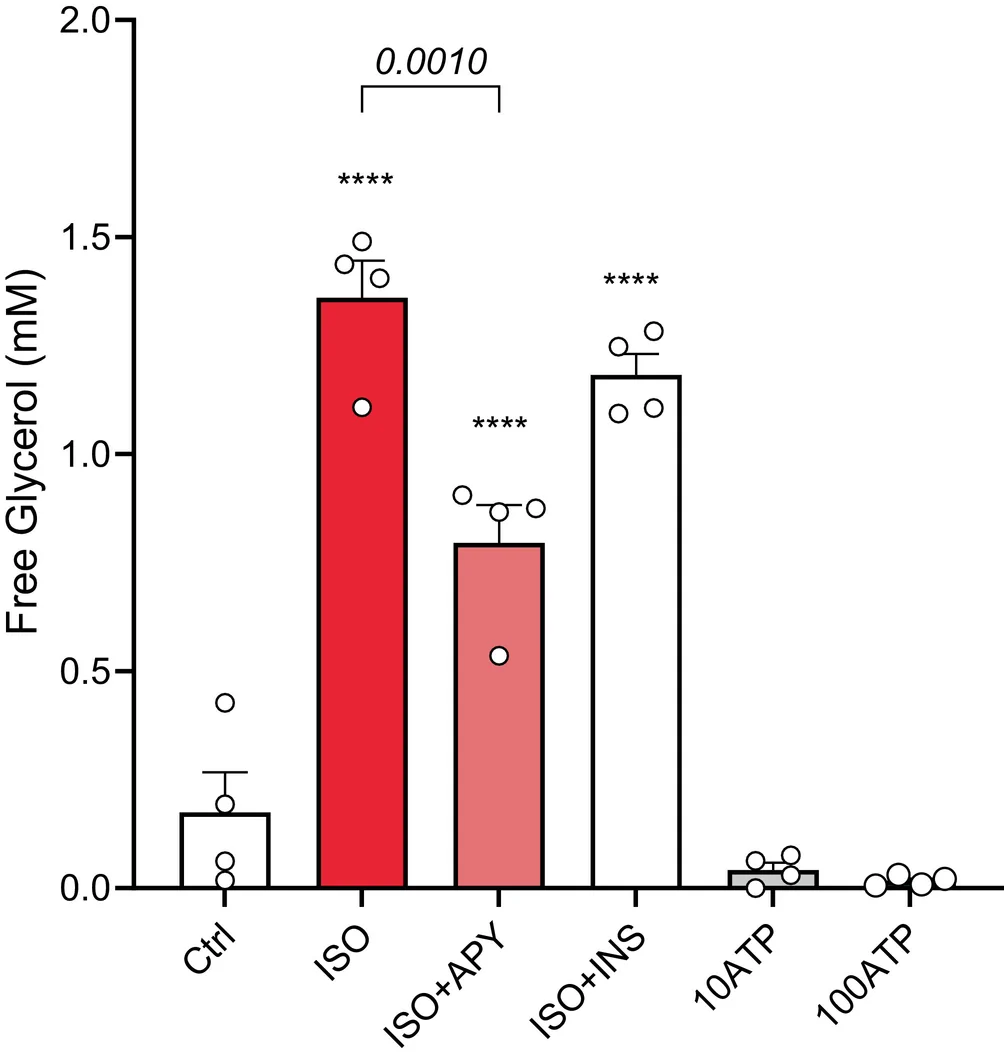
Glycerol release from mature adipocytes. Mature adipocytes were stimulated with adipolysis assay buffer (Ctrl), 10 μM FSK, 1 μM ISO, and ISO in combination with 5 U/mL APY, 1 μg/mL insulin (INS), and ATP (10 or 100 μM) alone. Abbreviations are the same as in the previous graphs. Data represent the mean ± SEM of 4 independent experiments (with 3 replicates per experiment). Significant *p* values are indicated in the graph. Asterisks depict significant differences between the control and tested compounds (****p < 0.0001), and *p* values show significant differences between ISO stimulation and ISO + test compounds.

In the next set of experiments, we investigated whether eATP impacts β‐adrenergically induced lipolysis, monitored by glycerol secretion. Stimulating cells with ATP alone had no significant effect on glycerol release (Figure 5). ISO increased glycerol release significantly, and this effect was reduced by pre‐treatment with apyrase. The observation that apyrase inhibited ISO‐stimulated lipolysis indicates that eATP might play a role in supporting lipolysis in these cells. Insulin tended to decrease isoproterenol‐induced lipolysis; however, the effect did not reach statistical significance.

### 2.6 Signaling Proteins and Calcium Signals

**FIGURE 6:**
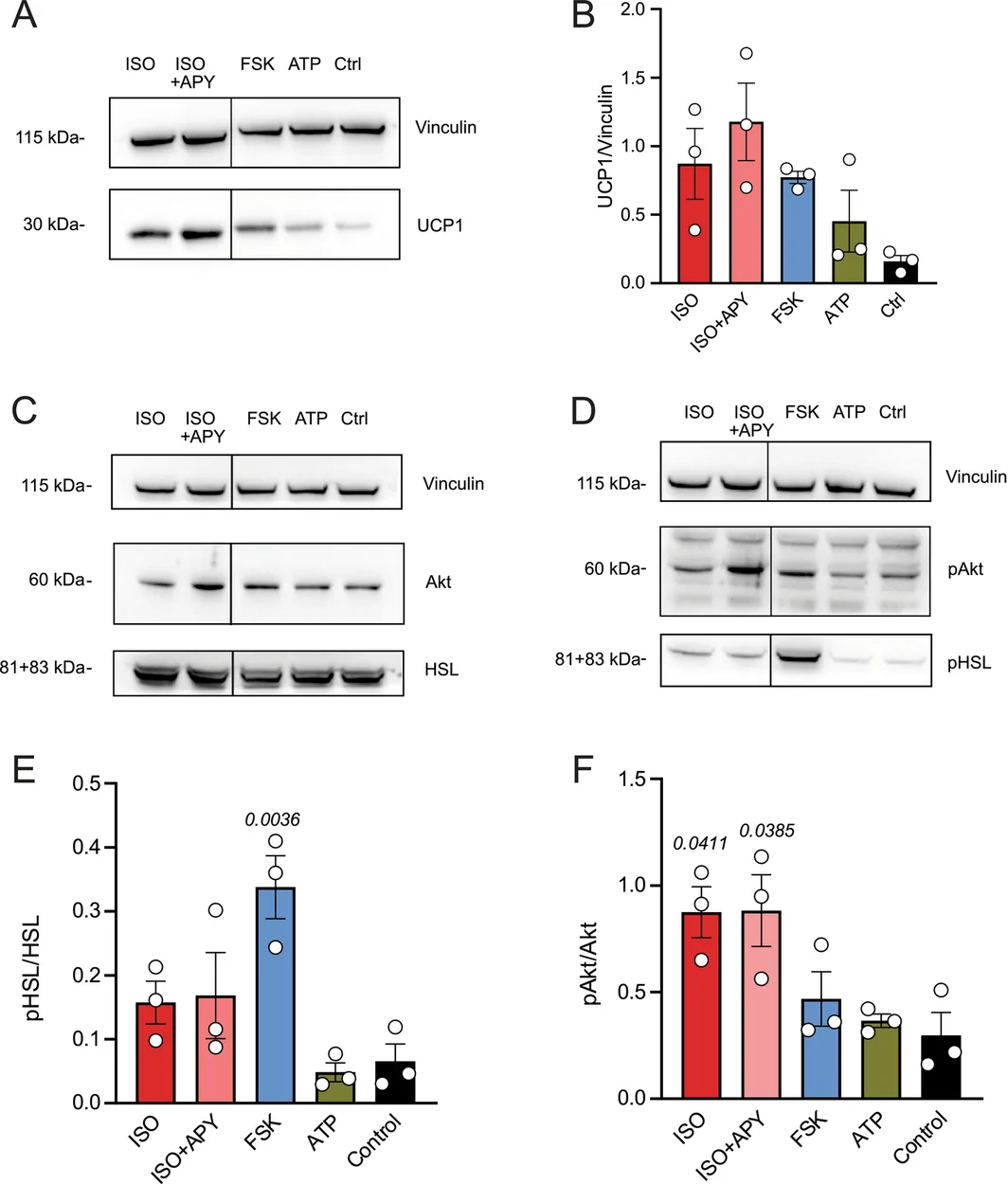
Content and phosphorylation of key proteins in mature brown adipocytes ± stimulation with purinergic agents. (A, C, D) Representative western blots of uncoupling protein‐1 (UCP1), Akt, hormone‐sensitive lipase (HSL), and phosphorylated forms pAKT and pHSL. Vinculin was used as a loading control in all. Quantification analysis of (B) UCP1/Vinculin; (E) pHSL (Ser^660^ phosphorylation)/HSL protein; and (F) pAkt (Ser^473^ phosphorylation)/Akt protein (middle band around 60 kDa was quantified). BA were stimulated with ISO ± apyrase or FSK, and ATP, or not stimulated, serving as controls (Ctrl). Protein and phosphorylation levels were compared to controls, and significant *p* values are indicated in the graph.

The next objective was to measure whether eATP, added exogenously or released from BA, affects calcium signaling and key signaling and lipolysis‐related proteins. The content of these proteins was determined by western blot analyses (Figure 6). It appeared that there was an increased content of UCP1 protein with all stimulations compared to control, and exogenous ATP alone seemed to have the least effect (Figure 6A). However, no significant differences between treatments were detected (Figure 6B). We further assayed levels of total and phosphorylated levels of Akt and hormone‐sensitive lipase (HSL), with phosphorylated forms indicative of activation. HSL^Ser660^ phosphorylation (pHSL) was highest in cells stimulated with forskolin compared to control (Figure 6C,D,E), consistent with the importance of cAMP signaling downstream of β‐adrenergic receptors. In contrast, Akt^Ser473^ phosphorylation (pAkt) was highest in ISO‐stimulated cells, indicating the important role of β‐adrenergic receptor signaling (Figure 6C,D,F). Notably, neither exogenous eATP nor apyrase had significant effects on levels of pHSL or pAkt compared to the respective controls.

**FIGURE 7:**
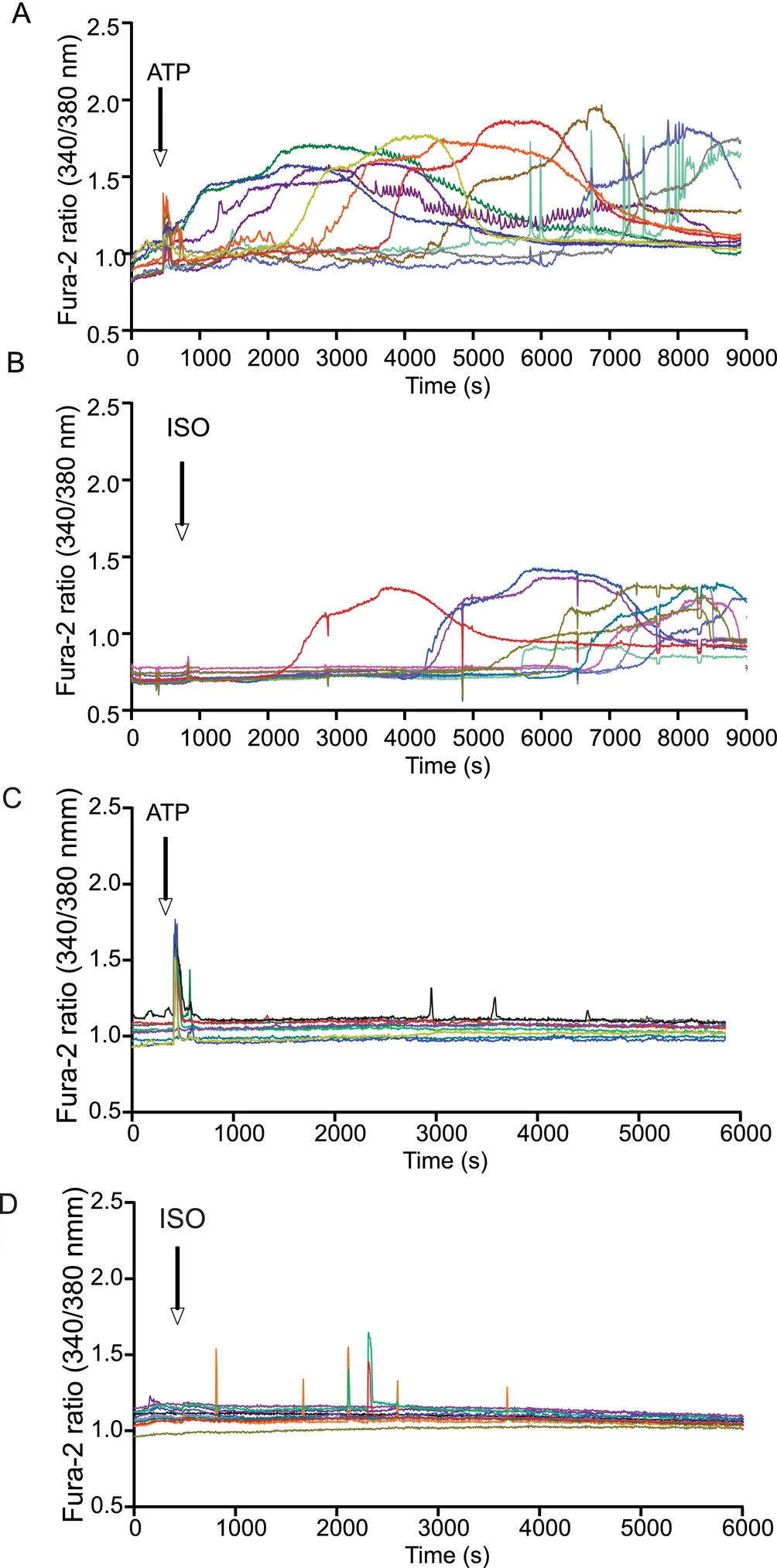
Effect of isoproterenol and ATP on calcium signals. (A, B) Representative responses in intracellular Ca^2+^ levels in mature adipocytes as determined from Fura‐2 ratios. Each colored line shows the response of one cell; altogether 10 cells from one experiment are shown. Cells were stimulated with 1 μM ISO (A) or 10 μM ATP. Three independent experiments were performed. (C, D) Representative responses in intracellular Ca^2+^ signals in pre‐mature adipocytes as determined from Fura‐2 ratios. Each colored line shows the response of one cell; altogether 10 cells in an experiment. Three independent experiments were performed. Cells were stimulated with 1 μM ISO (A) or 10 μM ATP.

Several P2 receptors have been described in both white and brown adipocytes [6]. Therefore, in another set of experiments, we tested the hypothesis that Ca^2+^ signals are involved in ISO‐stimulated events in BA. We monitored Ca^2+^ responses by means of Fura‐2 fluorescence. Consistently, exogenously applied eATP induced substantial both fast and slow Ca^2+^ signals, indicating involvement of both ligand‐gated ion channels (P2X) and G proteins coupled receptors (P2Y) (Figure 7A). Notably, ISO also induced Ca^2+^ signals, but with a substantially delayed onset (Figure 7B). In contrast, in pre‐adipocytes, ISO did not elicit any consistent Ca^2+^ signals, although addition of exogenous ATP induced a few transient events (Figure 7C,D). These data indicate that in mature BA, ATP release (induced by ISO) mediates autocrine (and potentially paracrine) ATP effects on purinergic receptors, which evoke Ca^2+^ signals consistent with activation of P2X and P2Y receptors.

### 2.7 Expression of Purinergic Receptors in Brown Adipocytes

Several purinergic receptors could mediate the observed cellular Ca^2+^ signals (Figure 7). To elucidate which receptors might be considered in potentiation of β‐adrenergic effects, we performed RNA‐sequencing on mature BA (WT‐1) treated with norepinephrine (NE, 1 μM) or vehicle (ctr). BA express significant levels of ionotropic P2X receptors (P2X3, P2X4, P2X5) and low levels of P2X6, P2X7 and metabotropic P2Y receptors (P2Y_2_, P2Y_6_) (Table 1, right). For completeness, we also included adenosine receptors (A_1_, A_2A_, A_2B_, A_3_), of which A1 is expressed at the highest level. Interestingly, NE appears to substantially increase expression of the P2X4 receptor and modestly increase levels of P2X3, P2X7, P2Y_2_ receptors, and A_2B_ receptors, and decrease expression of P2X5, P2X6, and P2Y_6_ receptors (Table 1, right). Moreover, expression of P2X5 and P2X7 receptors is increased in mature BA compared with pre‐adipocytes (Figure 1F).

**TABLE 1:** Purinergic and adenosine receptors—RNA‐seq (TPM values) in BAT and BA.

	iBAT 30°C	iBAT 16°C	iBAT 6°C	WT‐1 ctr	WT‐1 NE
Ion channel P2X receptors					
*P2rx1*	0.47	0.28	0.24	0.06	0.07
*P2rx2*	0.01	0.00	0.00	0.01	0.01
*P2rx3*	0.49	0.50	0.56	7.09	8.45
*P2rx4*	8.51	9.33	11.38	15.46	28.00
*P2rx5*	18.68	18.27	21.91	9.27	7.60
*P2rx6*	1.79	5.48	6.83	0.20	0.12
*P2rx7*	0.61	0.39	0.43	0.14	0.21
Metabotropic P2Y receptors					
*P2ry1*	4.38	2.48	2.64	0.00	0.02
*P2ry2*	1.10	2.43	3.11	0.56	0.64
*P2ry4*	0.00	0.00	0.00	0.00	0.00
*P2ry6*	0.96	1.06	0.75	0.22	0.05
*P2ry12*	0.50	0.13	0.14	0.00	0.00
*P2ry13*	0.08	0.13	0.08	0.01	0.00
*P2ry14*	1.15	0.64	0.68	0.00	0.00
Adenosine receptors					
*Adora1*	19.69	20.07	21.23	28.66	20.80
*Adora2a*	5.01	5.74	3.54	0.07	0.05
*Adora2b*	0.12	0.10	0.09	0.24	0.36
*Adora3*	0.00	0.04	0.01	0.00	0.00

*Note:* Expression profiles of purinergic receptors in BAT from five wild‐type mice housed at 30°C, 16°C, and 6°C. RNA‐seq was performed on interscapular brown adipose tissue (iBAT), and transcripts per million (TPM) values are given as average values for 5 mice. Expression profiles of purinergic receptors in WT‐1 cells untreated control (ctr) or treated with norepinephrine (NE). Results of 2 independent experiments, each with 4 replicates.

### 2.8 Effect of Temperature on Purinergic Receptor Expression in Mouse BAT

To elucidate purinergic receptor regulation in vivo, we performed RNA‐sequencing on interscapular BAT (iBAT) isolated from mice adapted to different environmental temperatures: 30°C (thermoneutrality), 16°C (mild cold) and 6°C (cold). Table 1 shows the complete data sets for expression of P2X and P2Y receptors as well as adenosine receptors. Expression profiles of P2 receptors in iBAT are partly similar to those in BA. Notably, P2X3 and P2X4 expression is lower in iBAT, while P2X5, P2X6, and P2X7 expression is higher in iBAT compared to BA. Expression of P2Y_1_, P2Y_12_, P2Y_14_, and A_2A_ is observed in iBAT, but is very low in BA.

**FIGURE 8:**
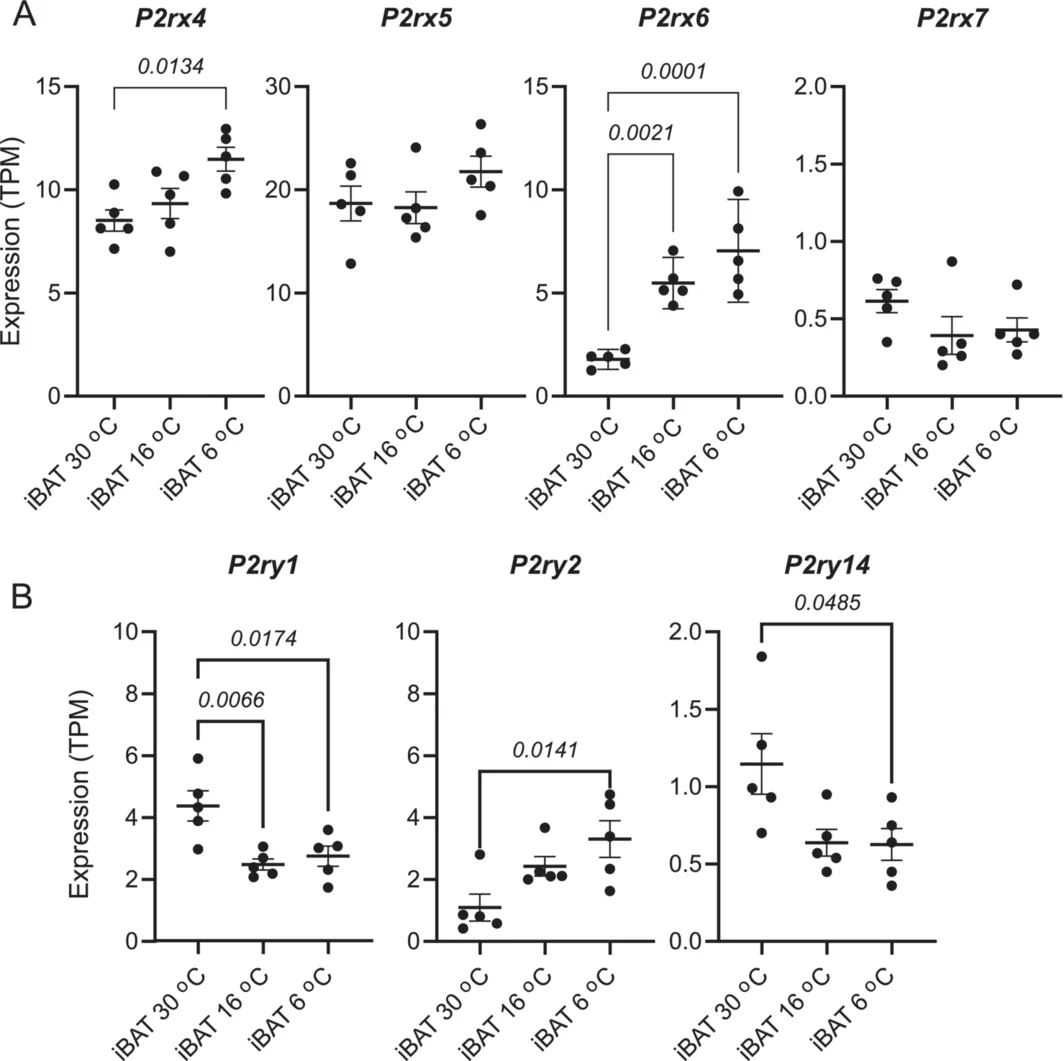
Effect of ambient temperature on expression of purinergic receptors in BAT of mice. RNA‐seq data shown as transcripts per million (TPM) mean values ± SEM in BAT dissected from mice kept in different environmental temperatures—30°C, 16°C and 6°C. (iBAT 30, 16 and 6, respectively). (A) Shows expression of ionotropic P2X receptors. (B) Expression of metabotropic P2Y receptors. Significant *p* values are indicated in the graph. Full data sets for P2X, P2Y and adenosine receptors are given in Table 1.

Figure 8 shows data for P2 receptors selected based on previous studies and/or receptors for which environmental temperature impacted expression levels. Interestingly, P2X5 receptor expression, although high, was not affected by temperature. In contrast, mRNA levels of P2X4 and P2X6 receptors were increased at low temperatures. The P2X7 receptor mRNA level tended to decrease, but the decrease did not reach statistical significance. Another receptor that was increased with low temperature was the P2Y_2_ receptor. Interestingly, other receptors utilizing Ca^2+^ signaling (P2Y_1_ receptors) and cAMP signaling (P2Y_14_ receptors) were downregulated at low temperatures.

## 3 Discussion

In this study, we investigated the mechanism and role of ATP release in brown adipocytes stimulated with β‐adrenergic agonists. We found that mature BA, but not pre‐adipocytes, release ATP in response to β‐adrenergic stimulation, and this involves Panx1, the level of which increases during differentiation of BA. β‐Adrenergic stimulation increases oxygen consumption, glycolysis, and lipolysis in BA, and these processes are sensitive to eATP levels and insulin. Exogenously added eATP on its own has only significant effects on glycolysis and, importantly, on Ca^2+^ signals. Below, we discuss these findings and propose that purinergic signaling, via ATP release and P2 receptor stimulation, tunes and optimizes the β‐adrenergic effects on cellular functions of BA. Furthermore, we discuss the expression and distribution of P2 receptors in BA and BAT.

In some studies mentioned in the introduction, off‐line eATP measurements were performed on BA or BAT [8, 12, 21]. Here, we have employed sensitive and quantitative methods to monitor eATP in an online assay on living cells, which enabled us to reveal new details about regulation of ATP release in BA. The β‐adrenergic receptor agonist ISO (and forskolin), but not the α‐adrenergic receptor agonist phenylephrine, increased eATP in BA (Figure 1). As a Panx1 inhibitor decreased ISO‐ and FSK‐induced eATP and because Panx1 is located on the plasma membrane of mature BA, we propose that the recorded eATP increase is due to ATP release via this activated channel. Notably, pre‐adipocytes have low Panx1 expression, and accordingly, they do not release ATP in response to either ISO or FSK. This is consistent with another study performed on the BA cell line iBAC showing that β_3_‐adrenergic receptors and apoptotic stimuli (UV light) activate Panx1 via the Gβγ subunit of the heterotrimeric G protein, and that Panx1 deficiency results in defective cold‐induced thermogenesis [12]. However, in contrast to our study, the authors suggest that these Panx1 effects were independent of ATP release and purinergic signaling.

The present observation that ATP release in BA increased with increased extracellular glucose is in line with similar effects seen in WA and pancreatic β‐cells [9, 22]. The present data on BA also show that the β‐adrenergic receptor, adenylyl cyclase, cAMP and PKA signaling are important for ATP release in BA (Figure 2). First, PKA inhibition reduced ATP release. Second, insulin, which activates PDEs [19, 20], decreased ATP release markedly, and PDE3 inhibition restored ATP release (Figure 2). Interestingly, these processes are similar in mature WA (C3H10T1/2 cells and freshly isolated murine adipocytes), although in those cells it is the α‐adrenergic receptors that stimulate ATP release [9]. We propose that an adrenergic receptors/cAMP/PKA/Panx1 axis is a conserved and functional signaling module in both white and brown adipocytes.

Notably, exogenous ATP, that is, experimentally applied ATP, had direct stimulatory effects on ECAR and Ca^2+^ signals (Figures 4, 7). As expected, oxygen consumption (OCR), ECAR and lipolysis (glycerol release) were strongly stimulated by β‐adrenergic receptor activation (Figures 3, 4, 5). Nevertheless, the observation that apyrase (the eATP scavenger), and possibly Panx1 inhibitor, reduced OCR, ECAR and glycerol release indicates that released eATP improved, or was permissive for, the optimal effect of the β‐adrenergic stimulation. The finding that addition of exogenous ATP on top of adrenergic stimulation did not affect OCR (or ECAR) is most likely because BA cells were already saturated with ATP released by themselves, which stimulated P2 receptors.

ECAR can have several components—lactic acid secretion, proton extrusion or bicarbonate import (via, e.g., MCT1,4, NHE1 transporter and NBCn1) [23] following metabolic CO_2_ production and ATP breakdown etc. However, since BA have very high lactate flux activity [23, 24], we propose that ECAR is a reasonable estimate of glycolysis in BA.

It should be noted that adenylyl cyclase stimulation with forskolin had smaller effects on OCR and ECAR than isoproterenol and was not affected by apyrase and Panx1 inhibitor (Figures 3, 4), although forskolin induced prominent ATP release (Figure 1). This indicates that the stimulation of the β‐adrenergic receptor, not only activation of adenylyl cyclase, is crucial for the full physiological effect. In one study, it is suggested that following β‐adrenergic receptor stimulation, Gβγ activates Panx1 [12]. However, other hitherto unexplored signaling and/or β‐adrenergic receptor/purinergic receptor interactions at the plasma membrane could be involved.

One of the remarkable observations pertains to the effect of insulin. Insulin appeared to reduce OCR and ECAR in isoproterenol‐stimulated BA (Figures 3, 4). As insulin activates PDE3 [19, 20] and reduces ATP release (Figure 2), one could speculate that reduced eATP signaling could tune down the effects of β‐adrenergic stimulation. However, in the basal state, insulin alone stimulated OCR and ECAR, likely by promoting glucose uptake and glycolysis. Notably, as we proposed for WA, also for BA we put forward the hypothesis that insulin could prevent excessive pathophysiological release of ATP and stimulation of low‐affinity P2X7 receptors in BA and other cells in adipose tissue, such as macrophages [9, 25]. Thus, diminished insulin sensitivity and resulting high eATP would promote inflammation in both WAT and BAT.

Glycerol release is a marker of lipolysis, that is, breakdown of triglycerides to glycerol and fatty acids (FA), where FA may activate the uncoupling capacity of UCP1 [1]. The finding that apyrase reduced the ISO effect on lipolysis indicates that eATP released by adrenergic stimulation had a permissive effect on lipolysis in BA, which is in accordance with a previous report on WA [9].

Perhaps the most convincing evidence that eATP co‐regulates BA function are the Ca^2+^ imaging experiments on mature BA. First, addition of exogenous ATP leads to Ca^2+^ increases in BA and the diverse Ca^2+^ responses most likely reflecting heterogeneity expression of P2 receptor subtypes, for example, P2X and P2Y [26], showing components of the Ca^2+^ influx and Ca^2+^ release (Figure 7). Compared to addition of exogenous ATP, ISO caused delayed Ca^2+^ responses, that occurred about the same time as the ATP release (Figure 1). The role of Ca^2+^ signaling in β‐adrenergically stimulated BA has been unresolved. It has been reported that β_3_‐adrenergic receptor stimulation led to Ca^2+^ release from mitochondria (a relatively slow process), though another study proposed that high basal Ca^2+^ influx was inhibited by β‐adrenergic stimulation [27, 28]. We propose that the most likely scenario is that ISO signaling induces ATP release (Figure 1), which via autocrine/paracrine stimulation of P2 receptors on BA triggers Ca^2+^ signals (Figure 7). In pre‐adipocytes, ISO did not induce Ca^2+^ signals, as only occasional Ca^2+^ activity was observed, while exogenously added ATP elicited fast Ca^2+^ responses in some pre‐adipocytes, indicating the presence of some P2 receptors. Previous studies by Pappone and co‐workers [26, 29] showed that rat BA expressed P2 receptors and that eATP simulated Ca^2+^ responses and increased basal heat production consistent with the presence of P2Y_2_, P2Y_6_ and P2Y_12_ receptors. In addition, the membrane capacitance changes indicating exocytosis were consistent with P2X receptors, but no clear agonist/antagonist profile could identify which of the receptors, that is, P2X1‐P2X7, were most involved. In fact, Pappone et al. [30] suggested that it is likely that sympathetic activation of thermogenic responses in rat BA normally has both adrenergic and purinergic components. Our study supports this original hypothesis.

Further support for involvement of purinergic signaling in thermogenic processes comes from our RNA‐sequencing analysis of BAT from mice exposed to 30°C, 16°C, and 6°C ambient temperatures (Figure 8). Interestingly, the mRNA levels of P2X4, P2X6, and P2Y_2_ receptors were increased at low temperatures in iBAT. These receptors may be of relevance as they are also expressed in BA (Table 1). The P2Y_2_ receptor is a high‐affinity Ca^2+^ signaling receptor that is widely distributed and has many roles [6, 11, 31], possibly in autocrine physiological function in BA. Also, future research on P2X receptors in BA will need to elucidate whether the P2X4 receptor is important in the plasma membrane or intracellular compartments, whether the P2X6 receptor can form functional homomers, or whether it requires other receptors, for example, the P2X4 receptor, to play a role at the cell surface [32, 33]. Furthermore, the role of the P2X3 receptor, which has high affinity for ATP and seems to be sensitive to norepinephrine in BA, needs to be explored.

The P2X5 receptor has been accepted as a marker of BAT, though in a European population the protein is truncated and dysfunctional due to one SNP [34]. In mice and rats, the P2X5 subunits are fully formed, though the outcome of a few published animal studies is varied, as outlined in the introduction. In our study, we found high expression of the P2X5 receptor in both BA and iBAT, though its expression was not influenced by low temperature.

Interestingly, in a recent study, it was proposed that stressed BA released ATP in response to imbalanced thermogenic activation, for example, UCP1‐deficiency or combined treatment with etomoxir and β_3_‐adrenergic activation, but not to β_3_‐adrenergic activation alone [21]. This treatment in mice caused higher expression and activation of P2X4 and P2X7 receptors on BAT‐resident macrophages. Activation of these purinergic receptors in macrophages drove BAT inflammation and dysfunctional thermogenesis [21]. A similar interplay between excessive ATP release from WA, macrophage P2X7 receptor stimulation, and inflammation was proposed earlier [9, 25]. Nevertheless, BA also express relatively high levels of high‐affinity P2X4 receptors that seem to be upregulated with norepinephrine, and they express lower levels of the P2X7 receptor, which is regarded as a danger signal sensor of high and sustained eATP (see above) [32]. However, this receptor can also be activated by acute low levels of eATP and stimulate aerobic glycolysis in cancer cells (Warburg effect), thus providing energy to support cell proliferation [32, 35, 36]. In our study on BA, we observed that exogenous ATP on its own increased ECAR slightly (Figure 4), possibly upregulating glycolysis via the P2X7 receptor (Figure 1F, Table 1).

In BA, as in WA [9], adrenergic stimulation leads to activation of Panx1, release of ATP, and eATP acting locally in an autocrine/paracrine manner on P2Y receptors, allowing release of Ca^2+^ from intracellular stores and Ca^2+^ influx through P2X receptors/ion channels and possibly through Panx1 channels. This reasoning is in line with higher Panx1 expression and higher ATP release in mature adipocytes (Figure 1). Notably, Panx1 can also be activated by several other receptors, post‐translational modifications, protein–protein interactions, cleavage of the C‐terminus [37], and thermal stress [12, 38]. This could explain that in β‐less mice (β1,2,3‐adrenergic receptor triple knockouts), mild cold exposure still induced increased UCP1 expression and improved metabolic function in BAT [5]. Interestingly, the mild cold stimulus (22°C) in β‐less mice showed increased expression of P2X7 and P2Y_12_ but not P2X5 receptors in BAT. In our study on wild‐type mice, we did not observe any significant change in the mRNA levels of these three receptors in response to a temperature decrease from 30°C to 16°C or 6°C (Figure 8, Table 1).

Summarizing our expression studies on BA and iBAT, the observed norepinephrine and/or cold‐induced thermogenic response on the P2Y_2_, P2X3, P2X4, and P2X6 receptor expression indicates that these P2 receptors could play a role in the response to cold.

In conclusion, the present study on BA shows that β‐adrenergic stimulation causes release of ATP through Panx1 channels, and the released ATP supports the autocrine and physiological effects of β‐adrenergic stimulation on oxygen consumption, extracellular acidification, and lipolysis. We have profiled expression of purinergic receptors in cultured brown adipocytes with or without norepinephrine stimulation, and in BAT at three different temperatures representing thermoneutrality, mild, and strong cold. We propose that activation of purinergic receptors mediates the impact of extracellular ATP on β‐adrenergic stimulation, possibly through Ca^2+^ signaling. We cannot resolve which P2 receptors are most important in BA, though expression signatures in BA and multicellular iBAT indicate that P2X3, P2X4, P2X6, and P2Y_2_ may be of importance in physiological regulation of BA. However, important functions of P2X5 and P2X7 receptors cannot be ruled out, as well as an interplay between different cell types in BAT. Another important finding is that insulin regulates ATP release from BA, possibly preventing excessive ATP release, thus providing a novel framework for understanding the role of insulin in BAT physiology and pathophysiology in the context of inflammation and type‐2 diabetes.

## 4 Materials and Methods

### 4.1 Cell Culture

All standard chemicals were purchased from Sigma‐Aldrich unless stated otherwise. The murine immortalized brown pre‐adipocyte cell line WT‐1 was kindly provided by C. Ronald Kahn [39]. WT‐1 cells were propagated in Dulbecco's Modified Eagle's Medium (DMEM) (Thermo Fisher Scientific) supplemented with 25 mM D‐Glucose, 10% fetal bovine serum (FBS) (Gibco, 10270 106), and 1% penicillin/streptomycin (P/S). Cells were cultured in a humidified atmosphere at 37°C with 5% CO_2_ and kept proliferating at sub‐confluent density. Prior to differentiation, WT‐1 cells were grown to 100% confluency, and from the day of confluence no P/S was included in the medium. Two days post‐confluence, designated day 0, the cells were induced to differentiate in medium containing 5 μg/mL insulin (Roche, 11376497001), 0.5 μM rosiglitazone (Cayman Chemical, 71740), 1 μM dexamethasone (Sigma‐Aldrich, D1756), and 0.5 mM methyl isobutyl xanthine (Sigma‐Aldrich, I5879). On day 2, cells were refreshed with DMEM containing 5 μg/mL insulin and 0.5 μM rosiglitazone. From day 4, the cells were cultured in DMEM basal medium only supplemented with 10% FBS and refreshed every other day. The cells were fully differentiated into mature adipocytes, ready for harvest and experimentation from day 6.

### 4.2 Western Blotting

Proteins were extracted from both WT‐1 pre‐ and mature adipocytes. The cells were grown and differentiated in 6‐well plates and treated with 1 μM isoproterenol, 10 μM forskolin or 100 μM ATP. Some cells were treated with 5 U/mL apyrase for 30 min before treatment with isoproterenol. The cells were incubated at 37°C with 5% CO_2_ for 3 or 6 h before lysate preparation. Samples were reduced by 5 min heating at 98°C in the presence of 50 mM dithiothreitol. Proteins (35 μg) were loaded on 10% polyacrylamide precast gels (Invitrogen, NP0302) and blotted onto PVDF membranes (Invitrogen, LC2005). Membranes were blocked with Tris‐buffered saline with 0.1% Tween 20 detergent buffer (TBST) and 5% skim milk at 37°C for 1 h. Thereafter they were incubated overnight at 4°C with primary antibodies against pannexin‐1 (1:1000 rabbit polyclonal, Alomone ACC‐234), vinculin (1:1000 mouse monoclonal, Sigma‐Aldrich V9131), β‐Actin (1:1000, Sigma‐Aldrich A3853), UCP1 (1:1000, Abcam ab10983), HSL (1:1000, Cell Signaling 4107S), HSL Ser^660^ phosphorylation (1:1000, Cell Signaling 4126S), Akt (1:1000, Cell Signaling 9272) and Akt S^743^ phosphorylation (1:1000, Cell Signaling 9271). The blots were incubated with appropriate secondary HRP‐conjugated antibodies (1:2000, Dako P0260 or Santa Cruz Biotechnology, sc‐2357) and developed with EZ‐ECL (Biological Industries). The intensity of the emitted light was visualized as bands by Fusion FX (Vilber Lourmat, Eberhardzell) and the band intensity was determined using the Image J software (U.S. National Institutes of Health).

### 4.3 Immunocytochemistry

Pre‐ and mature WT‐1 were trypsinized and allowed to attach to glass coverslips in a 6 well plate for 48 h. Cells were washed in PBS and fixed with 4% paraformaldehyde, permeabilized with 0.1% TritonX‐100 in 1% BSA for 12 min, washed and blocked with 2% BSA in PBS for 1 h. Cells were then incubated with antibodies against pannexin‐1 (2–4 μg/mL, HPA016930, Atlas antibodies) overnight at 4°C and then with a secondary antibody conjugated to Alexa 488 (1:400, Molecular Probes). DAPI (1:400, Molecular Probes) was used as a nuclear stain. For F‐actin staining, coverslips were incubated with Texas Red‐X Phalloidin or Alexa 647‐conjugated anti‐actin (1:40) for 20 min. Control slides were made without primary or secondary antibodies. Fluorescence was examined in a Leica SP 5X MP confocal laser scanning microscope with 40× 1.3 NA or 63× 1.4 NA objectives. Images were collected in sequential scans and analyzed in Leica LAS AF software.

### 4.4 ATP Release

Extracellular ATP (eATP) was monitored using luciferin/luciferase luminescence reaction in the extracellular milieu as previously described [9]. Briefly, fully differentiated cells were plated (80 000 cells/well) on gelatine‐coated 96‐well COSTAR white plates (Greiner, GR‐655088) and grown in 5.5 or 25 mM glucose 24 h before the experiment. Cells were gently washed and allowed to rest in 65 μL of physiological buffer containing (in mmol/L): 140 NaCl, 1 MgCl_2_, 1.5 CaCl_2_, 0.4 KH_2_PO_4_, 1.6 K_2_HPO_4_, 10 HEPES, and 5.5 glucose, pH 7.4. After 30 min, 25 μL of luciferin/luciferase mix (BioThema, ATP kit SL144‐041) was added, and the baseline was recorded for 9–14 min. It was imperative that the baseline was low, indicating that cells were not disturbed and already releasing ATP. Cells were then gently stimulated with 25 μL physiological buffer (control), 10 μmol/L forskolin (FSK) (Sigma‐Aldrich F6886), 1 μmol/L isoproterenol (ISO) (Sigma‐Aldrich, I5627), 5 μmol/L phenylephrine (Sigma‐Aldrich, P6126), 1 μmol/L ionomycin (Sigma‐Aldrich, I0634). In some experiments cells were pre‐incubated 45 min with 1 μg/mL of insulin, pannexin‐1 inhibitor 10Panx (100 μmol/L, Tocris, 3348/1), H‐89 (20 μmol/L, Calbiochem, 371963), Staurosporine (10 nmol/L, Sigma‐Aldrich, J4400), oligomycin (1 μmol/L, Sigma‐Aldrich, 75351), Cilostazol (50 μmol/L, Sigma‐Aldrich, C0737) or Rolipram (20 μmol/L, Sigma‐Aldrich, R6520). Effective concentrations of compounds were chosen in accordance with our earlier study on adipocytes and recommendations from manufacturers.

Live‐cell measurements were performed at 37°C using the Varioskan LUX multimode microplate reader, measuring every 3 min for 15 rounds. All experimental conditions were measured with either three or four replicates. For each experimental protocol, a standard curve was made with ATP concentrations ranging from 4.95 × 10^−9^ M to 3.3 × 10^−7^ M (ATP standard, BioThema). Cell numbers were determined using the cell counting kit‐8 (CCK‐8, Sigma‐Aldrich, 96992) in parallel wells with the same cell seeding. The relative luminescence was converted to nanomolar concentrations, determined from the standard curve. Finally, the concentration of eATP was corrected to 10^6^ cells/mL. Quantification of ATP release was shown as the difference between the basal extracellular ATP before stimulation and the peak value after addition of the indicated agonists. In some graphs, relative eATP levels between treatments are shown after the calibration was performed.

### 4.5 Seahorse Respirometry

Real‐time measurements of OCR and ECAR were performed using the Seahorse XF96 Extracellular Flux Analyzer (Seahorse Bioscience). Two days before the experiment, fully differentiated WT‐1 cells were plated (15 000 cells/well) on gelatin‐coated XF96 cell culture microplates. A day before the experiment, the sensor cartridges were hydrated in XF96 Calibrant (200 μL/well) and incubated overnight in the Seahorse Prep Station at 37°C with ambient CO_2_. On the day of the experiment, the media was changed from DMEM to Seahorse medium containing 5.5 mM glucose. The cell culture microplates were transferred to the Seahorse Prep station and incubated at 37°C with ambient CO_2_ for 1 h. In some experiments, cells were pre‐incubated for 45 min with the following test substances: apyrase, ^10^Panx, insulin or ATP. OCR and ECAR were measured at basal conditions for 5 measurements (33 min), followed by injection of Seahorse media containing 5.5 mM glucose (Ctrl), 1 μM isoproterenol, 10 μM forskolin, 10 μM or 100 μM ATP. Lastly, the mitochondrial stress test was performed according to the manufacturer's instructions and previous optimization on WT‐1 cells and [24] included following compounds were added successively: Oligomycin (1 μM), carbonyl cyanide‐4 (trifluoromethoxy) phenylhydrazone (FCCP, 1 μM), and a mixture of Rotenone and Antimycin A (Rot/AA, 1 μM). Quantifications were performed according to the manufacturer's instructions (Agilent Technologies). Seahorse experiments were performed in replicates of 4–6, and the mean of replicates was normalized to the stimulant injection (5th measurement), that is, the baseline. The area under the curve (AUC) of OCR was calculated after the addition of test compounds (from the 5th to the 15th measurement, 70 min), and the baseline was subtracted. The acute responses in ECAR were calculated at the end of the stimulation period (15th measurement) just before addition of Oligomycin. In addition, non‐normalized basal OCR and ECAR (5th measurement) was calculated to address possible effects of pre‐incubation with different compounds prior to stimulation.

### 4.6 Glycerol Release

WT‐1 mature adipocytes (day 6) were plated (80 000 cells/well) on 96‐well plates. After 24 h, cell culture medium was replaced with 100 μL/well of Adipolysis Assay Buffer and allowed to rest at 37°C with 5% CO_2_ for 4 h. Cells were then supplied with fresh Adipolysis Assay Buffer; some wells were also supplied with 5 U/mL apyrase and incubated for 30 min. Then, cells were stimulated with agonists (10 μM forskolin, 1 μM isoproterenol, 10 μM ATP or 100 μM ATP) for 6 h. All test conditions were performed in triplicates. Finally, supernatants from each well were collected and stored at −20°C. Glycerol release was measured according to the manufacturer's instructions using the Adipolysis Assay Kit (Sigma‐Aldrich, MAK117) and measurements were performed in the Varioskan LUX multimode microplate reader (Thermo Scientific).

### 4.7 Calcium Signals

WT‐1 cells were grown and differentiated in glass‐bottom dishes (Wilko, KIT‐3512). Adipocytes were loaded with Fura‐2 AM (3 μmol/L) for 30 min in physiological buffer with 5.4 mmol/L glucose, gently washed and mounted in the temperature‐regulated chamber (37°C). Experiments were conducted in standing‐bath configuration to avoid mechanical disturbance. After 5 min rest in a bath, cells were stimulated with ATP 10 μmol/L or isoproterenol (1 μmol/L) and lastly 1 μmol/L ionomycin. Changes in intracellular Ca^2+^ were followed in Nikon Eclipse Ti microscope with 40× NA1.4 objective. Fura‐2‐loaded cells were illuminated at λex = 340 nm for 60 ms and λex = 380 nm for 60 ms at 1 s intervals using a TILL Polychrome monochromator. Emission λem was collected at 500 nm by an image‐intensifying, charge‐coupled device (CCD) camera (Andor X3 897) and digitized by FEI image processing system (Thermo Fischer Scientific). LA Live Acquisition software was used to control the monochromator and the CCD camera. The intracellular Ca^2+^ was presented as the ratio of Fura‐2 fluorescence signals recorded at 340/380 nm.

### 4.8 Gene Expression in WT‐1 Cells

In the first series of experiments with pre‐adipocytes and mature WT‐1 cells, RNA was extracted using RNeasy Mini Kit (Qiagen) according to the manufacturer's instructions. DNA synthesis and RT‐qPCR cycling conditions were conducted as described [40] except that we used BIO‐RAD CFX384 Optics Module qPCR system and adjusted the total volume of each reaction to 10 μL. Each reaction contained 1.5 μL template cDNA, 5 μL of 2*SensiFAST SYBR Lo‐ROX mix (Bioline) and 0.2 pmol forward and reverse primers. Reactions were carried out in 384‐well plates in duplicates by using the following cycling 131 conditions: 95°C for 10 min, then 40 cycles of 95°C for 15 s, 55°C for 30 s and 72°C for 15 s, 132 followed by a melt curve from 65°C to 95°C with an increment of 0.5°C for 5 s, then 60°C for 30 s. Gene expression was normalized to the expression of TATA‐binding protein (Tbp). Primers used: *Tbp* (m), fwd‐ACCCTTCACCAATGACTCCTATG, rev‐ ATGATGACTGCAGCAAATCGC; *Ucp1* (m), fwd‐AGCCGGCTTAATGACTGGAG, rev‐ TCTGTAGGCTGCCCAATGAAC; *P2rx5* (m), fwd‐ATCATCCCCACAGTCATCAAC, rev‐ TGTCTCGGTAAAACTCGCTCT; *P2rx7* (m), fwd‐AAGGTCAAAGGCATAGCAGAG, rev‐ GTCCTAACTTCGTCACCCCA; *Panx1* (m), fwd‐TGTGGATTCATACTGCTGGG, rev‐ GCAGTAGGATGTAGGGGAAGA.

In the second series of experiments, we used RNA sequencing on mature WT‐1 cells treated with norepinephrine (1 μM) or vehicle (control) for 6 h.

### 4.9 In Vivo Mouse Study

C57BL/6 male mice were obtained from Taconic Biosciences. The mouse study was approved by the Danish National Committee for Animal Studies (2019‐15‐0201‐01674). Care and handling of mice were in accordance with local institutional recommendations. Mice were housed at 22°C for 8 weeks, 6 weeks at 30°C, followed by a gradual temperature decline to 6°C. Interscapular brown adipose tissue (BAT) was harvested from five mice at each of the three temperatures, 30°C, 16°C and 6°C.

### 4.10 RNA Sequencing, and Bioinformatics

RNA from either BAT or mature WT‐1 brown adipocytes was isolated using Direct‐zol RNA MiniPrep (Zymo Research). The quantity of extracted RNA was determined with Qubit RNA BR Assay Kit (ThermoFisher) and the RNA Integrity number (RINe) on the 4200 TapeStation system (Agilent Technologies) with High Sensitivity RNA Assay Kit (Agilent Technologies), respectively. For mRNA enrichment, a total of 300 ng of total RNA for each sample was processed using Dynabeads mRNA Purification Kit (ThermoFisher). RNA library preparation was made with MGIEasy RNA Directional Library Prep Set (MGI Tech Co. Ltd.) following the manufacturer's instructions. Concentration and fragment analysis on prepared libraries were assessed using Qubit 1X dsDNA HS Assay Kit (ThermoFisher) and Agilent High Sensitivity D1000 Assay Kit (Agilent Technologies). Pooled and concentrated libraries were sequenced with 2 × 100 bp paired‐end reads on DNBSEQ‐G400 (MGI Tech Co. Ltd.) using the DNBSEQ‐G400RS High‐throughput Sequencing Set (MGI Tech Co. Ltd.) according to the manufacturer's instructions. Quality control of raw reads was performed using SOAPnuke (v2.1.5) to obtain clean reads. Clean reads were aligned to the reference genome GRCm39 by Bowtie2 (v2.4.5). Gene expression quantification was conducted with RSEM (v1.3.3), generating expected read counts and transcripts per million (TPM) values for each gene.

### 4.11 Statistics

GraphPad Prism (10.0.2) was used for graphics and statistical analyses. Data were tested for normality. Data are shown as raw data, mean ± standard error of the mean (SEM), and data normalized to controls. Normalized data were analyzed with One‐sample *t*‐tests (unpaired, two‐tailed), followed by correction for multiple comparisons with the Bonferroni method. Remaining data were analyzed with *t*‐tests or One‐way analysis of variance (ANOVA) with a Dunnett's multiple comparisons test to evaluate the effect of different treatments compared to the control, or the Tukey's method when different treatments were compared. A significance level of *p* < 0.05 was chosen, and the significant *p* values and/or asterisks are indicated in the graphs.

## Author Contributions

**Marco Tozzi:** conceptualization, investigation, methodology, data curation, formal analysis, writing – original draft, writing – review and editing, validation, visualization. **Emilie B. Berggreen:** investigation, writing – original draft, methodology, validation, visualization, formal analysis, data curation, writing – review and editing. **Rui Zhang:** investigation, methodology, data curation, formal analysis, writing – review and editing. **Anders Gudiksen:** investigation, writing – review and editing, data curation. **Wei Li:** investigation, writing – review and editing, formal analysis, data curation. **Mathias B. Matz:** investigation, data curation, validation. **Henriette Pilegaard:** investigation, writing – review and editing, supervision. **Jacob B. Hansen:** conceptualization, writing – original draft, writing – review and editing, supervision, resources. **Ivana Novak:** conceptualization, investigation, funding acquisition, writing – original draft, writing – review and editing, methodology, formal analysis, project administration, supervision, resources.

## Funding

This research was funded by the Independent Research Fund Denmark (grant numbers 4002‐00162B and 8020‐00254B).

## Conflicts of Interest

The authors declare no conflicts of interest.

## Data Availability

All data required to evaluate the conclusions reached in the paper are present in the paper.
